# Celecoxib Exerts Neuroprotective Effects in β-Amyloid-Treated SH-SY5Y Cells Through the Regulation of Heme Oxygenase-1: Novel Insights for an Old Drug

**DOI:** 10.3389/fcell.2020.561179

**Published:** 2020-09-29

**Authors:** Emanuela Mhillaj, Massimiliano Papi, Fabiola Paciello, Andrea Silvestrini, Rolando Rolesi, Valentina Palmieri, Giordano Perini, Anna Rita Fetoni, Luigia Trabace, Cesare Mancuso

**Affiliations:** ^1^Department of Healthcare Surveillance and Bioethics, Section of Pharmacology, Università Cattolica del Sacro Cuore, Rome, Italy; ^2^Fondazione Policlinico Universitario Agostino Gemelli IRCCS, Rome, Italy; ^3^Department of Neuroscience, Università Cattolica del Sacro Cuore, Rome, Italy; ^4^Department of Basic Biotechnological Sciences, Intensivological and Perioperative Clinics, Università Cattolica del Sacro Cuore, Rome, Italy; ^5^Department of Head and Neck Surgery, Università Cattolica del Sacro Cuore, Rome, Italy; ^6^Department of Clinical and Experimental Medicine, University of Foggia, Foggia, Italy

**Keywords:** Alzheimer’s disease, amyloid-β-peptide, bilirubin, carbon monoxide, cyclooxygenase, heme oxygenase, reactive oxygen species

## Abstract

The formation and aggregation of amyloid-β-peptide (Aβ) into soluble and insoluble species represent the pathological hallmarks of Alzheimer’s disease (AD). Over the last few years, however, soluble Aβ (sAβ) prevailed over fibrillar Aβ (fAβ) as determinant of neurotoxicity. One of the main therapeutic strategies for challenging neurodegeneration is to fight against neuroinflammation and prevent free radical-induced damage: in this light, the heme oxygenase/biliverdin reductase (HO/BVR) system is considered a promising drug target. The aim of this work was to investigate whether or not celecoxib (CXB), a selective inhibitor of the pro-inflammatory cyclooxygenase-2, modulates the HO/BVR system and prevents lipid peroxidation in SH-SY5Y neuroblastoma cells. Both sAβ (6.25–50 nM) and fAβ (1.25–50 nM) dose-dependently over-expressed inducible HO (HO-1) after 24 h of incubation, reaching statistical significance at 25 and 6.25 nM, respectively. Interestingly, CXB (1–10 μM, for 1 h) further enhanced Aβ-induced HO-1 expression through the nuclear translocation of the transcriptional factor Nrf2. Furthermore, 10 μM CXB counteracted the Aβ-induced ROS production with a mechanism fully dependent on HO-1 up-regulation; nevertheless, 10 μM CXB significantly counteracted only 25 nM sAβ-induced lipid peroxidation damage in SH-SY5Y neurons by modulating HO-1. Both carbon monoxide (CORM-2, 50 nM) and bilirubin (50 nM) significantly prevented ROS production in Aβ-treated neurons and favored both the slowdown of the growth rate of Aβ oligomers and the decrease in oligomer/fibril final size. In conclusion, these results suggest a novel mechanism through which CXB is neuroprotective in subjects with early AD or mild cognitive impairment.

## Introduction

Alzheimer’s disease (AD) is a neurodegenerative disorder characterized by progressive cognitive impairment, memory loss, inability to perform daily activities, and is the leading cause of dementia. The deposition of both senile plaques and neurofibrillary tangles, formed by the aggregation of fibrillar amyloid-β-peptide (fAβ) and hyperphosphorylated tau protein, respectively, are considered the pathological hallmarks of AD (Hardy and Selkoe, 2002). However, due to recent preclinical and clinical discoveries, such as the lack of correlation between senile plaque deposition and cognitive impairment or the therapeutic failure of drugs whose mechanism of action was aimed to reduce Aβ deposition or to increase its clearance, the involvement of fAβ in brain damage has been heavily questioned (Graham et al., 2017). Thus, significant research in the last decade has advanced a novel hypothesis that highlights the role of soluble forms of Aβ (sAβ), including the soluble oligomers produced during Aβ aggregation, as determinants of neurotoxicity (Selkoe and Hardy, 2016; Mhillaj et al., 2017). In this regard, a strong association has been shown between abnormal cerebral levels of sAβ forms and loss of synaptic plasticity (Wilcox et al., 2011; Park et al., 2013), inhibition of long-term potentiation (LTP) (Walsh et al., 2002), alteration of glutamatergic synapses (Green and LaFerla, 2008; Canas et al., 2014) and cognitive impairment (Tucci et al., 2014; Balducci et al., 2016; Mhillaj et al., 2018b). As far as the molecular mechanisms involved in AD, the strong and long-lasting neuroinflammatory response, together with the abnormal formation of reactive oxygen species and reactive nitrogen species (ROS and RNS, respectively), has been reported to be responsible for the increasing neuronal death, mainly in brain cognitive areas (e.g., hippocampus and frontal cortex) (reviewed in Agostinho et al., 2010). Incidentally, cyclooxygenase-2 (COX-2), by producing both free radicals and neuroinflammatory prostaglandins, plays a main role in AD pathogenesis and the administration of non-steroidal antinflammatory drugs (NSAIDs), including COX-2 inhibitors, has been considered a prophylactic approach to reduce the risk to develop AD (Hoozemans and O’Banion, 2005; Minghetti, 2007).

Heme oxygenase (HO) is a microsomal enzyme exerting important physiological functions through the biological activities of its metabolites. HO transforms hemoprotein’s heme moieties into ferrous iron, carbon monoxide (CO) and biliverdin (BV), this latter being further reduced into bilirubin (BR) by the cytosolic biliverdin reductase (BVR) (Maines, 1997). Two isoforms of HO have been identified, the first inducible (HO-1) under pro-oxidant conditions and the second constitutive (HO-2) involved in the physiologic turnover of heme (Maines, 1997). Over the last 25 years, several papers have been published demonstrating a marked induction of HO-1 in neurons and glial cells from AD brain and this phenomenon has been explained as an attempt of the neural tissue to react against oxidant/inflammatory damage by increasing the production of neuroprotectants, such as CO and BR (Schipper et al., 2006; Hettiarachchi et al., 2017; Nitti et al., 2018). Intriguingly, the over-expression of HO-1 has been also detected in lymphocytes and plasma from AD subjects, thus putting forth the hypothesis that HO-1 is a peripheral biomarker of AD (Calabrese et al., 2006; Di Domenico et al., 2012). Since HO-1 and BVR may undergo post-translational modifications in AD hippocampi which impair their enzymatic activities (Barone et al., 2011a,b, 2012b), a common strategy to preserve neuroprotection is to up-regulate HO-1 through the administration of some drugs (e.g., atorvastatin) or herb-derived antioxidants (e.g., ferulic acid, curcumin, rosmarinic acid, etc.) (Barone et al., 2012a; Butterfield et al., 2012; Catino et al., 2015; Fetoni et al., 2015; Mhillaj et al., 2018a, 2019). Although this huge amount of data, the vast majority of which obtained by analyzing *postmortem* brain specimens, only few papers have addressed the role played by sAβ or fAβ in the regulation of HO-1 and BVR with results not always comparable. Indeed, earliest studies did not focus on the differential effects of Aβ aggregation status, maybe because this issue was not considered interesting at that time.

On these premises, the first aim of this work is to fully characterize, by using a pharmacological approach, the differential regulation of the HO/BVR system by both sAβ and fAβ in the human neuroblastoma SH-SY5Y cells, a reliable experimental system widely used to study neurodegeneration and AD (Marrazzo et al., 2019; Wang et al., 2019; Celik et al., 2020). Furthermore, since *in vivo* data have shown that the COX-2 inhibitor celecoxib (CXB) reduces neuroinflammation and prevents cognitive impairment and behavioral abnormalities in sAβ-treated rats, the second aim of this work is to investigate whether or not CXB modulates the HO/BVR system in SH-SY5Y cells, thus widening the *spectrum* of its therapeutic activity.

## Materials and Methods

### Chemicals

Celecoxib was purchased from Tocris (BioTechne, Milan, Italy) and 1 mM stock solutions were prepared in DMSO. Bilirubin and Zinc-protoporphyrin-IX (ZnPP-IX, Frontier Scientific, Logan, UT, United States) were dissolved in alkaline aqueous solution. Tricarbonyldichlororuthenium (II) (CORM-2, Sigma-Aldrich, Milan, Italy) was dissolved in DMSO at the stock solution of 10 mM.

### Aβ Preparation and Aggregation Analysis

The Aβ_1__–__42_ peptide (hereafter referred to as Aβ) was purchased from Tocris (BioTechne, Milan, Italy). The soluble form of Aβ (sAβ) was obtained by dissolving the peptide in sterile distilled water at the concentration of 4 μM. For the fibrillary form (fAβ), the peptide was firstly dissolved in 100% hexafluoroisopropanol (HFIP) at the concentration of 4 μM and the solution evaporated to obtain the peptide film, as previously described (Stine et al., 2011). Then, the Aβ film was resuspended in DMSO, sonicated, diluted in 10 mM HCl and incubated at 37°C for 24 h.

For aggregation analysis, Aβ solutions were freshly prepared before each experiment by diluting either sAβ or fAβ in cell culture medium (see below) at the final concentrations of 25 nM and 6.25 nM, respectively; in selected experiments, both sAβ and fAβ were exposed to either 10 μM CXB or 50 nM BR or 50 nM CORM-2. Each sample was incubated at 37°C for 24 h under quiescent conditions and no significant changes in the pH of the solutions were detected over time. Residual DMSO from CXB or CORM-2 stock solutions did not interfere with Aβ aggregation.

Aβ aggregation status was structurally characterized by dynamic light scattering (DLS) using Zetasizer Nano ZS (Malvern, Herrenberg, Germany), as previously described (Palmieri et al., 2014). Solvent-resistant micro-cuvettes have been used for experiments using a fixed position (4.65 mm) with an automatic attenuator and at a controlled temperature (37°C). For each sample, 3 measurements were averaged.

Sample imaging was performed by atomic force microscopy (AFM), as previously described (Palmieri et al., 2017). Briefly, Aβ samples at fixed time points, were drop casted of fresh cleaved Mica disks and air dried. After sample preparation, measurements were immediately performed with a NanoWizard II AFM (JPK Instruments AG, Berlin, Germany) using silicon cantilevers with high aspect-ratio conical silicon tips (CSC36 Mikro-Masch, Tallinn, Estonia).

### Cell Culture

SH-SY5Y neuroblastoma cells were provided through the courtesy of Prof. Randall N. Pittman (Department of Pharmacology, University of Pennsylvania, Philadelphia, PA, United States) and cultured in Minimum Eagle’s Medium (MEM, Euroclone, Pero, Italy):F12 (Gibco, Life Tecnologies, Monza, Italy) supplemented with 1X non-essential aminoacids (Euroclone), 1 mM sodium pyruvate (Gibco), 1.5 g/L sodium bicarbonate, 1% penicillin/streptomycin (Euroclone) and 10% fetal calf serum (FCS, Euroclone), in a humidified incubator at 37°C and 5% CO_2_.

The day before the experiment, 1.2 × 10^6^ SH-SY5Y cells (10^th^–14^th^ passage) were seeded in 6-multiwell plates at a density of 120,000 cells/cm^2^. After overnight incubation, cells were treated with either sAβ (6.25–50 nM) or fAβ (1.56–50 nM) for 24 h. In another experimental setting, SH-SY5Y cells were treated with CXB (0.5–20 μM) for 1 h and then exposed to either cell culture medium or 25 nM sAβ or 6.25 nM fAβ for 24 h. To evaluate the effects of HO blockade, SH-SY5Y cells were treated with CXB plus sAβ or fAβ as above in the presence of ZnPP-IX (2.5 μM). Finally, in selected experiments, cells were incubated with either BR (50 nM) or CORM-2 (50 nM) for 6 h and then replaced with media containing sAβ (25 nM) or fAβ (6.25 nM) plus BR or CORM-2, for further 24 h. Drug dilutions were prepared in fresh culture medium immediately before performing the experiments. All the pharmacological manipulations were performed in triplicate. No significant changes in the pH of cell culture medium were detected after each treatment.

### Western Blot

Both HO-1 and HO-2 and BVR and β-actin levels in SH-SY5Y cells were detected by Western Blot as previously described (Catino et al., 2015). An anti-HO-2 antibody (1:1000, Stressgen, Enzo Life Sciences, DBA Italia, Segrate, Milan, Italy) was used. An anti-β-actin rabbit monoclonal antibody (1:1000; Stressgen) was used to detect β-actin. Nitrocellulose membranes were stripped and then re-probed with the anti-β-actin antibody to confirm equal protein loading. The HO-1 or HO-2 or BVR/β-actin ratios were calculated and expressed as a percentage compared to the control group.

### Immunofluorescence Analysis

Immunofluorescence for 4-hydroxynonenals (4-HNE) and nuclear factor erythroid 2-related factor 2 (Nrf2) have been performed as previously described (Catino et al., 2015). Briefly, 100,000 cells, seeded in glass coverslips (10 mm diameter), were fixed with 4% paraformaldehyde for 15 min at room temperature, permeated with 0.1% Triton-X for 15 min before being blocked in 0.3% BSA for 20 min. Samples were then incubated for 3 h with primary rabbit anti-4-HNE (Cat#HNE11-S, Alpha Diagnostic, Int., San Antonio, TX, United States) or mouse anti-Nrf2 (Abcam, Cambridge, MA, United States) antibody diluted 1:100 in 0.3% BSA in phosphate buffered saline (PBS). At the end of incubation, all samples were washed twice in PBS and incubated at room temperature for 90 min, light-protected, with secondary antibodies diluted 1:1000 in PBS. Goat anti-rabbit 488 (Alexa Fluor) was used for 4-HNE, whereas donkey anti-mouse 546 (Alexa Fluor) was used to detect Nrf2 labeling. Moreover, cell nuclei were counterstained with DAPI (1:1000 in PBS) for 10 min at room temperature, in a light-protected environment. Subsequently, the samples were coverslipped with an antifade medium (ProLong Gold; Invitrogen). Images (40×) were obtained with a confocal laser scanning system (Nikon Ti-E, Confocal Head A1 MP, Tokyo, Japan). A semi-quantitative analysis of fluorescence signals was quantified with ImageJ (version 1.51s); each evaluation was conducted on at least 15 fields randomly selected for each of the experimental conditions. Control experiments were performed by omitting the primary antibody during processing of tissue randomly selected across experimental groups (not shown).

### ROS Detection

The intracellular ROS were detected by fluorescence using the 2,7-dichlorofluorescein diacetate (DCFDA) Cellular ROS Assay kit (Abcam, Cambridge, MA, United States), according to the manufacturer’s instructions. Briefly, SH-SY5Y cells were seeded into 96-well plates at a cell density of 2.5 × 10^4^ cells/well and allowed to attach overnight. The day of the experiment, cells were washed and incubated with a freshly prepared solution of DCFDA (25 μM) at 37°C for 45 min in the dark. Immediately after, cells were washed and then incubated in 8-replicates for each of the established experimental protocols described above. Fluorescence signal was measured, with a reading time of 1 s, in a microplate reader (Victor3, Perkin Elmer, United States) with precision at 485 nm < 0.5% and temperature control at 37°C, set at an excitation wavelength of 485 nm and an emission wavelength of 535 nm. Data were expressed as percentage of control after background subtraction.

### Statistical Analysis

Analysis of the data were obtained by Graph Pad^®^ 6.0 software. Results are presented as mean ± standard error of the mean (SEM) of N replicates per group. Data sets have been analyzed by One-way ANOVA followed by a Tukey’s multiple comparison test. Differences were considered statistically significant at *P* < 0.05.

## Results

### Effects of Aβ on the HO/BVR System in SH-SY5Y Human Neuroblastoma Cells

**FIGURE 1:**
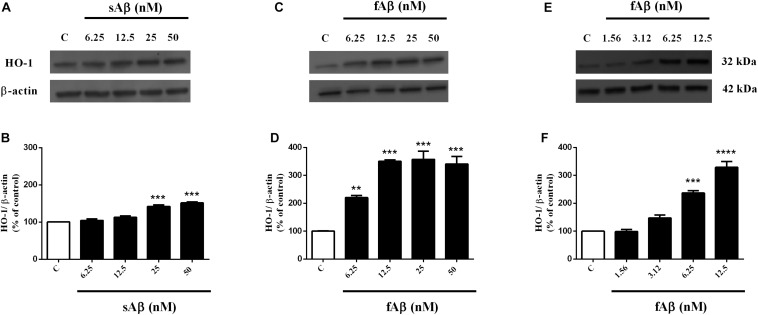
Soluble and fibrillar Aβ (sAβ and fAβ, respectively) increase heme oxygenase-1 (HO-1) expression in SH-SY5Y neurons. SH-SY5Y cells were treated with sAβ (6.25–50 nM) and fAβ (1.56–50 nM) for 24 h and Western Blot was performed as described in Section “Materials and Methods.” **(A,C,E)** Show representative gels regarding HO-1 protein expression following sAβ and fAβ treatment, as above. Bar graphs represent the quantification of HO-1 protein levels normalized for β-actin expression. Data are expressed as mean ± SEM of 3/4 replicates per group and analyzed by One-way ANOVA followed by Tukey’s test: ***P < 0.001 vs. C **(B)**; **P < 0.01 vs. C and ***P < 0.001 vs. C **(D)**; ***P < 0.001 vs. C and ****P < 0.0001 vs. C **(F)**. C, control.

**FIGURE 2:**
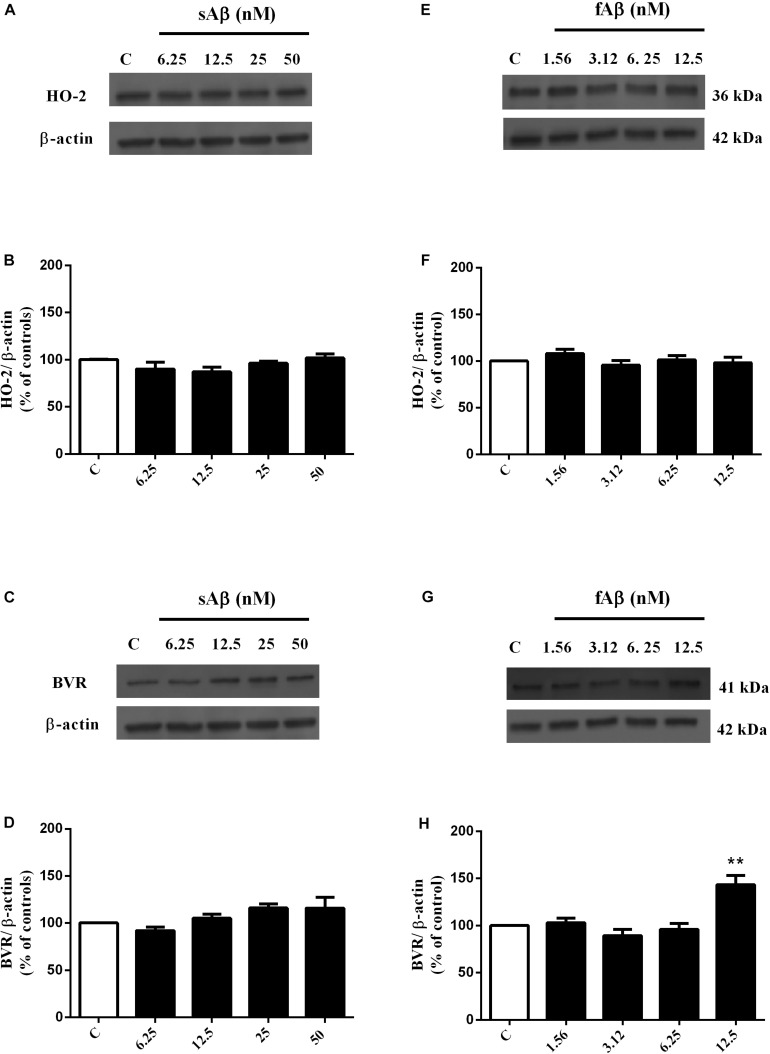
Effects of soluble and fibrillar Aβ (sAβ and fAβ, respectively) on heme oxygenase-2 (HO-2) and biliverdin reductase (BVR) expression in SH-SY5Y neurons. SH-SY5Y cells were treated with sAβ (6.25–50 nM) and fAβ (6.25–50 nM) for 24 h and Western Blot was performed as described in Section “Materials and Methods.” **(A,C,E,G)** Show representative gels regarding HO-2 and BVR protein expression following sAβ and fAβ treatment, as above. Bar graphs represent the quantification of HO-2 and BVR protein levels normalized for β-actin expression. Data are expressed as mean ± SEM of 4/5 replicates per group and analyzed by One-way ANOVA followed by Tukey’s test: **P < 0.01 vs. C **(H)**. C, control.

As shown in Figures 1A,B, sAβ (6.25–50 nM for 24 h) increased HO-1 expression and 25 nM was the lowest concentration reaching statistical significance (Figure 1B, One-way ANOVA followed by Tukey’s test, ^∗∗∗^*P* < 0.001 vs. C). Similarly, in a first set of experiments, the effect of fAβ on HO-1 expression was tested by using the same concentration-range and time of incubation used for sAβ (Figures 1C,D); however, by analyzing these data, a significant induction of HO-1 as low as 6.25 nM fAβ was detected (Figure 1D, One-way ANOVA followed by Tukey’s test, ^∗∗^*P* < 0.01 vs. C). This last result focused the attention on the possible effects of fAβ on HO-1 induction at concentrations lower than 6.25 nM and suggested widening the dose-range including lowest fAβ concentrations. Indeed, a dose-dependent increase in HO-1 expression as low as 1.56 nM fAβ was detected (Figures 1E,F), and this set of experiments confirmed 6.25 nM fAβ as the lowest concentration able to increase significantly HO-1 protein level (Figure 1F, One-way ANOVA followed by Tukey’s test, ^∗∗∗^*P* < 0.001 vs. C). With regard to HO-2 and BVR (Figures 2A–H), neither sAβ nor fAβ significantly modulated HO-2 expression, whereas only fAβ up-regulated BVR, reaching statistical significance at 12.5 nM (Figure 2H, One-way ANOVA followed by Tukey’s test, ^∗∗^*P* < 0.01 vs. C). The lowest effective concentrations of sAβ or fAβ for 24 h were used for further studies. It is noteworthy to mention that at time points shorter than 24 h of incubation, neither sAβ nor fAβ had any significant effect on HO-1, HO-2 and BVR protein expression (data not shown).

### Effects of CXB on Aβ-Mediated HO/BVR System in SH-SY5Y Human Neuroblastoma Cells

**FIGURE 3:**
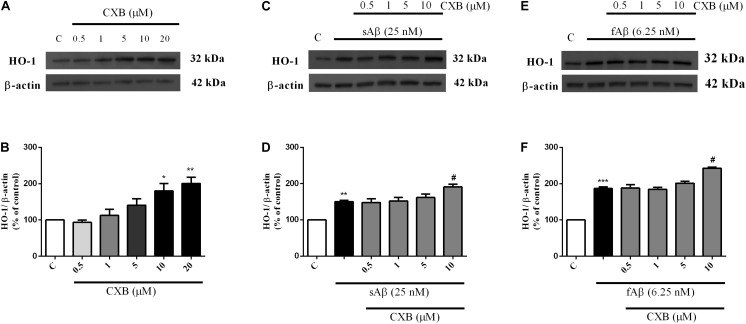
Celecoxib (CXB) effects on basal and Aβ-induced heme oxygenase-1 (HO-1) expression in SH-SY5Y neurons. SH-SY5Y cells were treated with CXB (0.5–10 μM) for 1 h and then incubated with cell culture medium for 24 h **(A)**; in another set of experiments, SH-SY5Y cells were treated with CXB (0.5–10 μM) for 1 h and then incubated with 25 nM sAβ or 6.25 nM fAβ for 24 h (**C,E**, respectively). Following incubation, cells were harvested and Western Blot was performed as described in Section “Materials and Methods.” **(A,C,E)** Show representative gels evaluating HO-1 protein expression. Bar graphs represent the quantification of HO-1 protein levels normalized for β-actin expression. Data are expressed as mean ± SEM of 4/5 replicates per group and analyzed by One-way ANOVA followed by Tukey’s test: *P < 0.05 and **P < 0.01 vs. C **(B)**; **P < 0.01 vs. C and ^#^*P* < 0.05 vs. sAβ **(D)**; ***P < 0.001 vs. C and ^#^*P* < 0.001 vs. fAβ **(F)**. C, control.

**FIGURE 4:**
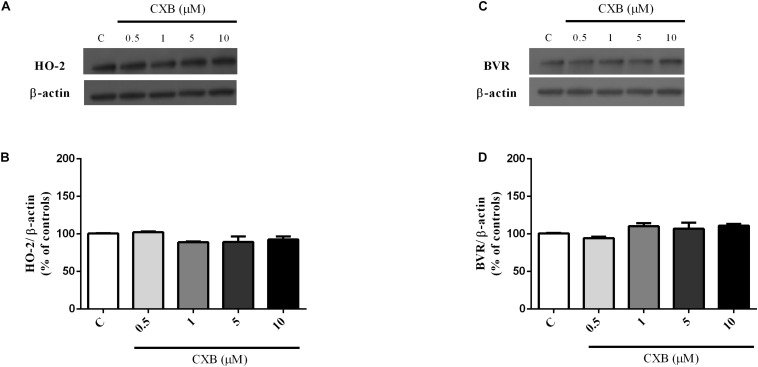
Celecoxib (FA) effects on basal heme oxygenase-2 (HO-2) and biliverdin reductase (BVR) expression in SH-SY5Y neurons. SH-SY5Y cells were treated with CXB (0.5–10 μM) for 1 h and then incubated with cell culture medium for 24 h. Following incubation, cells were harvested and Western Blot was performed as described in Section “Materials and Methods.” **(A,C)** Show representative gels evaluating HO-2 and BVR protein expression. Bar graphs represent the quantification of HO-2 and BVR protein levels normalized for β-actin expression. Data are expressed as mean ± SEM of 4/5 replicates per group and analyzed by one-way ANOVA followed by Tukey’s test: *P* > 0.05.

As shown in Figures 3A,B, CXB (0.5–20 μM for 1 h) dose-dependently over-expressed HO-1 in SH-SY5Y neurons, reaching statistical significance at 10 μM (Figure 3B, One-way ANOVA followed by Tukey’s test, ^∗^*P* < 0.05 vs. C). As far as the effect of CXB on Aβ-induced HO-1, Figures 3C–F, show as CXB (0.5–10 μM) potentiated 25 nM sAβ- and 6.25 nM fAβ- induced HO-1 up-regulation, respectively, reaching statistical significance at 10 μM (Figure 3D, One-way ANOVA followed by Tukey’s test, ^∗∗^*P* < 0.01 vs. C, **^#^***P* < 0.05 vs. sAβ; Figure 3F, One-way ANOVA followed by Tukey’s test, ^∗∗∗^*P* < 0.001 vs. C, **^#^***P* < 0.001 vs. fAβ). As shown in Figures 4A–D, no significant modulation of HO-2 and BVR protein levels by CXB have been detected (Figure 4B, One-way ANOVA followed by Tukey’s test *P* = 0.095; Figure 4D, One-way ANOVA followed by Tukey’s test *P* = 0.151).

**FIGURE 5:**
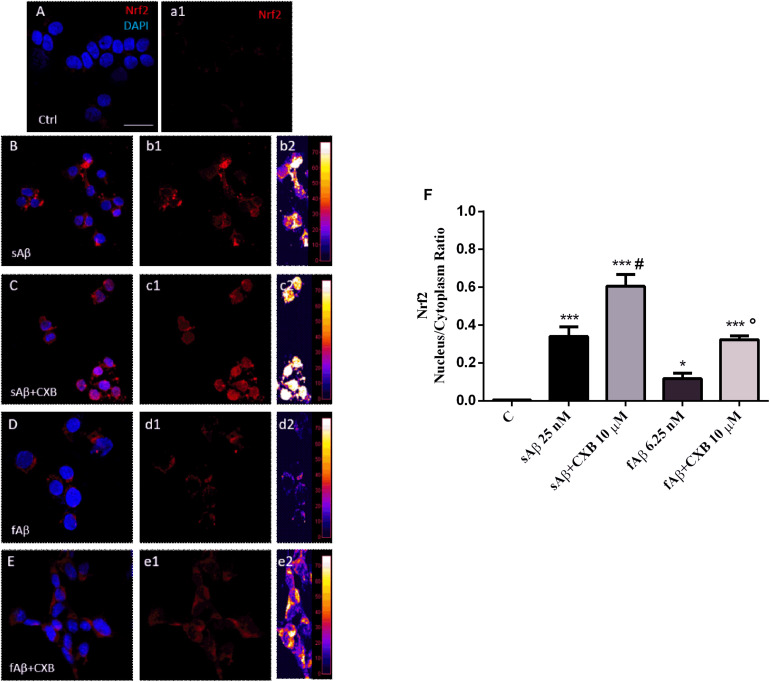
Nrf2 activation and translocation into the nucleus in SH-SY5Y neurons. **(A–E)** Representative images from three independent immunofluorescence experiments in which double-labeling with DAPI and anti-Nrf2 antibody **(a1–e1)** was performed in 25 nM sAβ- or 6.25 nM fAβ- treated SH-SY5Y neurons without or with 10 μM CXB, as described in Section “Materials and Methods.” Merged images are shown in **(A–E)**. **(b2–e2)** Images show the distribution of fluorescence intensity signal in a pseudo-color rainbow scale. Scale bar: 20 μm. **(F)** Represents the quantification of the Nrf2 nucleus/cytoplasm *ratio* in treated cells. Data are expressed as mean ± SEM of 11/13 replicates per group and analyzed by One-way ANOVA followed by Tukey’s test: ***P < 0.001 vs. C, *P < 0.05 vs. C, **^#^***P* < 0.001 vs. sAβ, °*P* < 0.01 vs. fAβ **(F)**. A.U., arbitrary units; C, Control.

A common mechanism through which HO-1 exerts neuroprotective effects in several cell types, including SH-SY5Y neurons, is the nuclear translocation of the transcriptional inducer Nrf2 (Johnson and Johnson, 2015). Therefore, the next step was to study whether CXB favors the Nrf2 cytosol-to-nucleus translocation in SH-SY5Y neurons. As shown in Figure 5, in neurons exposed to sAβ or fAβ, a faint-to-moderate Nrf2 translocation into the nucleus was detected, although some signal was confined into the cytoplasm (panels B-b2 and D-d2). Moreover, CXB induced a strong Nrf2 translocation into the nucleus, as reported by the fluorescence signal (panels C-c2 and E-e2). Notably, quantitative analysis of the Nrf2 nucleus/cytoplasm *ratio* revealed that 10 μM CXB increased Nrf2 nuclear translocation in both 25 nM sAβ- and 6.25 nM fAβ- treated cells (Figure 5F, One-way ANOVA followed by Tukey’s test, ^∗∗∗^*P* < 0.001 vs. C, ^∗^*P* < 0.05 vs. C, **^#^***P* < 0.001 vs. sAβ, **°***P* < 0.01 vs. fAβ), but CXB-induced Nrf2 nuclear translocation was greater in fAβ-exposed neurons (median fold change of sAβ+CXB/sAβ = 1.78 vs. fAβ+CXB/fAβ = 2.74), thus confirming the HO-1 induction detected in Figures 3C–F. Furthermore, these findings confirm that the exposure of SH-SY5Y cells to CXB is long enough to induce Nrf2 at the nuclear level and, presumably, to favor HO-1 over-expression during the 24 h incubation with either cell culture medium or sAβ or fAβ. Finally, these results mirror other findings in vascular endothelial cells and in macrophages, thus confirming how Nrf2-related transcription of cytoprotective genes in response to CXB is a conserved antioxidant mechanism (Wang et al., 2011; Al-Rashed et al., 2018). Incidentally, residual DMSO from CXB stock solution did not have any significant effect on protein levels and Nrf2 translocation.

### Characterization of Aβ Aggregation Forms

**FIGURE 6:**
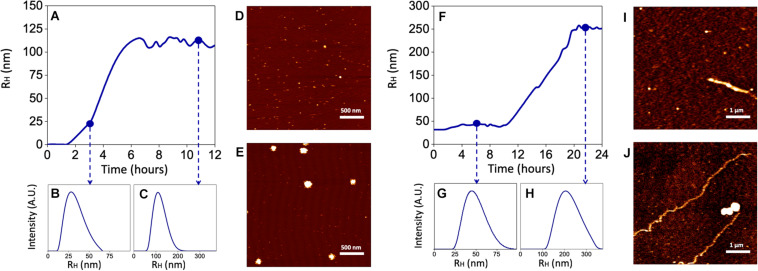
Kinetics of Aβ aggregation and oligomer/fibril formation by Dynamic Light Scattering (DLS) and Atomic Force Microscopy (AFM). 25 nM sAβ or 6.25 nM fAβ were incubated in cell culture medium at 37°C for 24 h under quiescent conditions. At fixed time points, Aβ samples were drop casted of fresh cleaved Mica disks and air dried. **(A–C)** Show a representative monomers aggregation kinetics of 25 nM sAβ, measured by DLS. **(D,E)** Representative AFM micrographs of early oligomers **(D)** and oligomers after 12 h of aggregation **(E)** are reported. **(F–H)** 6.25 nM fAβ aggregation kinetics, assessed by DLS, are reported. Representative AFM images of fibrils before **(I)** and after **(J)** aggregation are reported with monomers and oligomers coexisting with fibrils in the final phase.

Since both the rate of Aβ aggregation and size of oligomers/fibrils have been shown to vary depending on the concentration of the peptide and the buffer in which aggregation takes place (Nag et al., 2011; Nichols et al., 2015), these experiments have been carried out by using sAβ and fAβ at the lowest concentrations found effective to up-regulate HO-1, diluted in the cell culture medium and incubated at 37°C for 24 h under quiescent conditions. As shown in Figure 6A, 25 nM sAβ oligomerization starts after a lag-phase of ∼ 2 h, during which the peptide is prevalently in the monomeric form [hydrodynamic radius (RH) ∼ 1 nm], sharply increases within 6 h and reaches a plateau at ∼ 8 h. As early as 3 h, small oligomers with RH ∼20 nm prevail (Figure 6B), whereas at the plateau the oligomer size reaches RH ∼ 110 nm (Figure 6C). These results have been confirmed by AFM experiments showing the presence of a few monomers together with oligomers throughout the whole oligomerization process (Figures 6D,E).

With regard to 6.25 nM fAβ, the lag-phase before fibril elongation is ∼10 h: over this time, a few fibrils with RH ∼44 nm and 1–2 μm in length have been detected (Figures 6F,G,I). Conversely, after 10 h, the formation of longer fibrils takes place and reaches a plateau at ∼20 h: at this last time-point, longer fibrils with RH ∼267 nm and ∼10–15 μm in length have been detected (Figures 6H,J). Over the whole fibrillation period, Aβ oligomers, with maximal RH ∼150 nm have been also detected, implying a mixed population of Aβ soluble and insoluble species in these samples (Figures 6I,J).

### Effects of CXB on ROS Generation and Lipid Peroxidation in SH-SY5Y Cells

**FIGURE 7:**
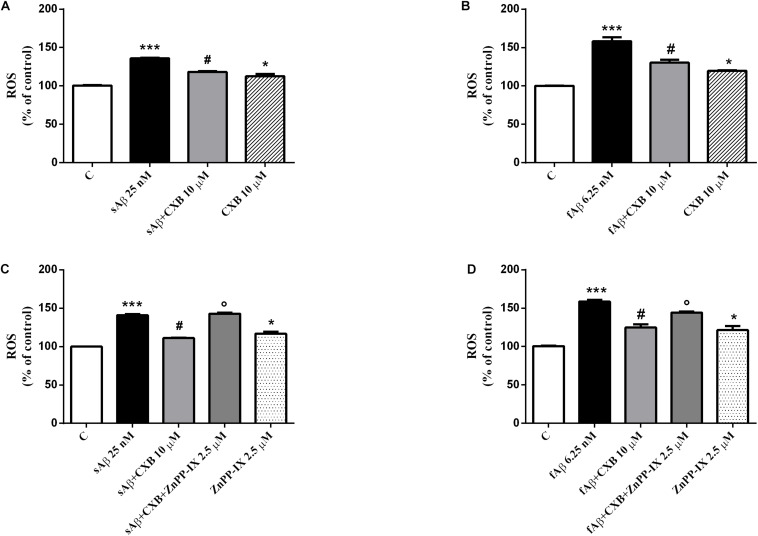
Celecoxib (CXB) contrasts Aβ-induced reactive oxygen species (ROS) formation through the HO activity in SH-SY5Y neurons. SH-SY5Y cells were treated with 10 μM CXB for 1 h and then incubated with either cell culture medium or 25 nM sAβ **(A)** or 6.25 nM fAβ **(B)** for 24 h. Following incubation, intracellular ROS cells were measured by fluorimetric detection as described in Section “Materials and Methods.” In selected experiments, SH-SY5Y neurons were incubated as above in the presence of 2.5 μM ZnPP-IX **(C,D)**. Data are expressed as a mean ± SEM of 12/14 replicates per group and analyzed by One-way ANOVA followed by Tukey’s test: ***P < 0.001 vs. C, ^#^*P* < 0.01 vs. sAβ, *P < 0.05 vs. C **(A,C)**; ***P < 0.0001 vs. C, ^#^*P* < 0.001 vs. fAβ, *P < 0.05 vs. C **(B,D)**; °*P* < 0.001 vs. sAβ+CXB **(C)**; °*P* < 0.05 vs. fAβ+CXB **(D)**. C, control.

As mentioned in Section “Introduction,” a common mechanism shared by sAβ and fAβ to induce neurodegeneration is the production of ROS, which in turn, up-regulate HO-1. Regarding the effect of CXB on Aβ-induced ROS production, data summarized in Figures 7A,B, demonstrate a significant antioxidant activity of 10 μM CXB against 25 nM sAβ and 6.25 nM fAβ, respectively, in SH-SY5Y neurons (Figure 7A, One-way ANOVA followed by Tukey’s test, ^∗∗∗^*P* < 0.001 vs. C, **^#^***P* < 0.01 vs. sAβ; Figure 7B, One-way ANOVA followed by Tukey’s test, ^∗∗∗^*P* < 0.0001 vs. C, **^#^***P* < 0.001 vs. fAβ). In this experimental system, 10 μM CXB alone weakly increased basal ROS production (Figures 7A,B, One-way ANOVA followed by Tukey’s test, ^∗^*P* < 0.05 vs. C).

To exclude a potential effect of CXB on Aβ aggregation as an adjuvant antioxidant mechanism, its effect on sAβ and fAβ aggregation has been studied. The results confirm the lack of any effect of 10 μM CXB on the aggregation of 25 nM sAβ and 6.25 nM fAβ over 24 h (data not shown).

Finally, in order to link the antioxidant effect of CXB with HO-1 over-expression, experiments with the HO-inhibitor ZnPP-IX have been performed according to current literature (Catino et al., 2015; Wang et al., 2016; Hui et al., 2018). As shown in Figures 7C,D, 2.5 μM ZnPP-IX fully counteracted 10 μM CXB-related ROS generation in SH-SY5Y cells treated with 25 nM sAβ, whereas the inhibitor only partially reverted ROS generation in 6.25 nM fAβ-exposed SH-SY5Y cells (Figure 7C, One-way ANOVA followed by Tukey’s test, °*P* < 0.001 vs. sAβ+CXB; Figure 7D, One-way ANOVA followed by Tukey’s test, °*P* < 0.05 vs. fAβ+CXB). Importantly, ZnPP-IX alone increased basal ROS production thus confirming the tonic antioxidant effect of HO-1 on SH-SY5Y cells (Figures 7C,D, One-way ANOVA followed by Tukey’s test, ^∗^*P* < 0.05 vs. C).

**FIGURE 8:**
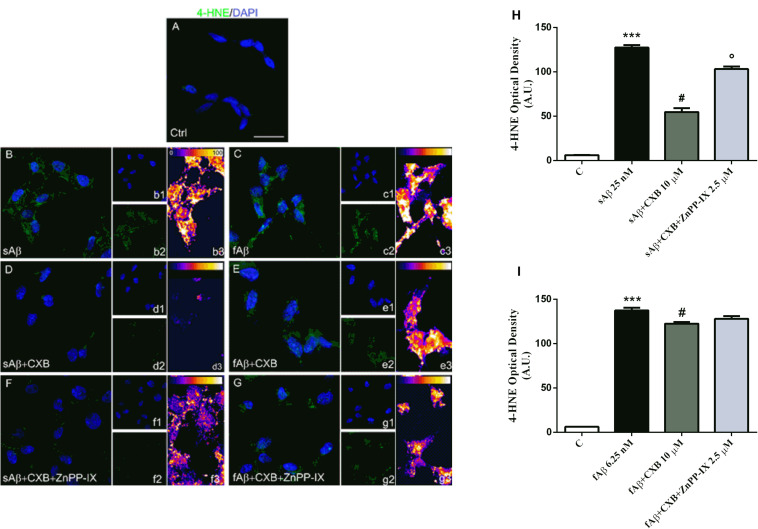
Celecoxib (CXB) counteracts Aβ-induced lipid peroxidation through the HO activity in SH-SY5Y neurons. **(A–G)** Representative images from three independent immunofluorescence experiments in which double-labeling with DAPI **(b1–g1)** and anti-4-HNE antibody **(b2–g2)** was carried out in SH-SY5Y neurons treated as described in the legend of Figure 5. Merged images are shown in **(A–G)**. **(b3–g3)** Images showing the distribution of fluorescence intensity signal in a pseudo-color rainbow scale. Scale bar: 20 μm. **(H,I)** Represent the quantification of 4-HNE fluorescence. Data are expressed as mean ± SEM of 15 replicates per group and analyzed by One-way ANOVA followed by Tukey’s test: ***P < 0.001 vs. C, **^#^***P* < 0.001 vs. sAβ, °*P* < 0.001 vs. sAβ+CXB **(H)**; ***P < 0.001 vs. C, **^#^***P* < 0.01 vs. fAβ **(I)**. A.U., arbitrary units; C, Control.

The differential effects of sAβ and fAβ on ROS production have been reflected on cell damage. As a biomarker of lipid peroxidation, 4-HNE have been assayed. As shown in Figure 8, 4-HNE labeling was faint in control cells (panel A), but increased markedly in cells treated with both sAβ (panels B-b3) and fAβ (panels C-c3), as also confirmed by fluorescence quantification (Figures 8H,I, One-way ANOVA followed by Tukey’s test, ^∗∗∗^*P* < 0.001 vs. C). Moreover, 10 μM CXB markedly reduced neuronal damage only in sAβ-treated cells (panels D-d3 and Figure 8H, One-way ANOVA followed by Tukey’s test, **^#^***P* < 0.001 vs. sAβ), whereas only a weak reduction in 4-HNE has been detected in neurons exposed to fAβ (panels E-e3 and Figure 8I, One-way ANOVA followed by Tukey’s test, **^#^***P* < 0.01 vs. fAβ). Finally, 2.5 μM ZnPP-IX significantly reverted CXB-related 4-HNE inhibition only in SH-SY5Y cells treated with sAβ (panel F-f3 and Figure 8H, One-way ANOVA followed by Tukey’s °*P* < 0.001 vs. sAβ+CXB). Residual DMSO from CXB stock solution did not have any significant effect on the results described above.

A possible mechanism through which CXB counteracts Aβ-induced ROS production and neurotoxicity is related to the ability of this drug to further increase Aβ induced-HO-1 up-regulation (Figures 3C–F), resembling a well-known mechanism involved in the neuroprotective effects of several agents under redox imbalance (Calabrese et al., 2008; Butterfield et al., 2012; Catino et al., 2015). In this frame, the inhibition of HO activity by ZnPP-IX confirms the main role played by HO-1 in CXB-related neuroprotection.

### HO By-products With Neuroprotective Properties

**FIGURE 9:**
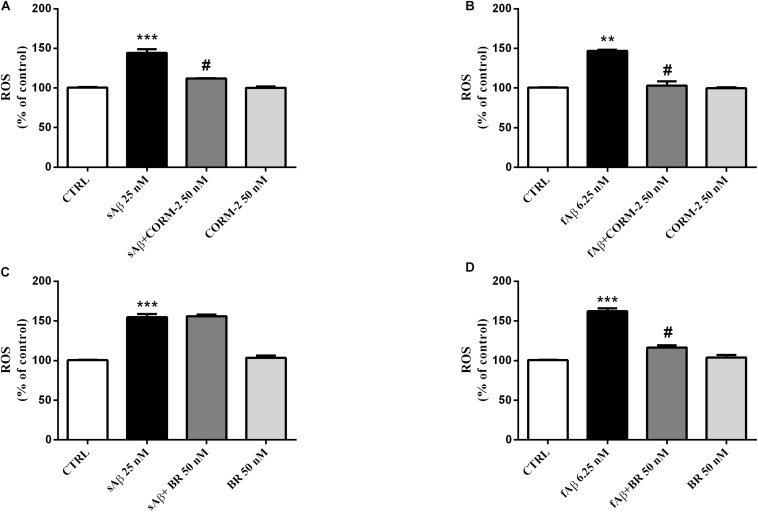
Antioxidant effect of the carbon monoxide donor CORM-2 and bilirubin (BR) in SH-SY5Y neurons exposed to Aβ. SH-SY5Y neurons were treated with 50 nM CORM-2 or 50 nM BR alone or in the presence of either 25 nM sAβ or fAβ for 24 h. At the end of incubation, intracellular ROS cells were measured by fluorimetric detection as described in Section “Materials and Methods.” Data are expressed as a mean ± SEM of 12/13 replicates per group and analyzed by One-way ANOVA followed by Tukey’s test: ***P < 0.001 vs. C, ^#^*P* < 0.01 vs. sAβ **(A)**; **P < 0.01 vs. C, ^#^*P* < 0.01 vs. fAβ **(B)**; ***P < 0.001 vs. C **(C)**; ***P < 0.001 vs. C, ^#^*P* < 0.01 vs. fAβ **(D)**. C, control.

The experiments described above demonstrated the main involvement of HO activity on CXB-mediated antioxidant effects. The next step was to identify which, among the HO by-products, is involved in the antioxidant effects previously described. The major concern we had to face with, while designing these experiments, was the choice of both BR and CORM-2 (a CO donor) concentrations so that they could not result toxic when co- administered with either sAβ or fAβ. In order to solve this issue, BR and CORM-2 were both used at 50 nM, a concentration found safe for SH-SY5Y neurons (Dal-Cim et al., 2012; Catino et al., 2015). As shown in Figures 9A,B, 50 nM CORM-2 significantly reduced ROS production stimulated by both 25 nM sAβ and 6.25 nM fAβ, respectively (Figure 9A, One-way ANOVA followed by Tukey’s test, ^∗∗∗^*P* < 0.001 vs. C, **^#^***P* < 0.01 vs. sAβ; Figure 9B, One-way ANOVA followed by Tukey’s test, ^∗∗^*P* < 0.01 vs. C, **^#^***P* < 0.01 vs. fAβ), whereas 50 nM BV did not have any effect (data not shown). On the contrary, 50 nM BR significantly inhibited only 6.25 nM fAβ-induced ROS production (Figure 9C, One-way ANOVA followed by Tukey’s test, ^∗∗∗^*P* < 0.001 vs. C, Figure 9D, One-way ANOVA followed by Tukey’s test, ^∗∗∗^*P* < 0.001 vs. C, **^#^***P* < 0.01 vs. fAβ).

**FIGURE 10:**
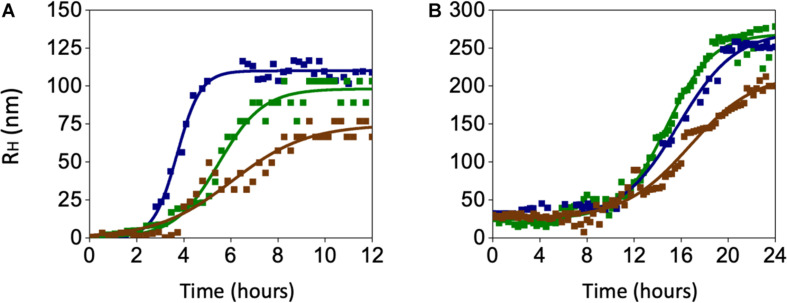
Effects of carbon monoxide donor CORM-2 and bilirubin (BR) on the kinetics of Aβ aggregation. **(A)** A representative aggregation kinetics, measured by DLS, of 25 nM sAβ (blu squares) in the presence of 50 nM CORM-2 (green squares) or 50 nM BR (red squares) is reported. **(B)** A representative aggregation kinetics, measured by DLS, of 6.25 nM fAβ (blu squares) in the presence of 50 nM CORM-2 (green squares) or 50 nM BR (red squares) is reported (see section “Materials and Methods” for further details).

In search for alternate mechanisms involved in CO and BR neuroprotective effects, specific experiments to assess their interaction with Aβ were performed. As shown in Figure 10 and Table 1, both 50 nM CORM-2 and 50 nM BR prolonged the lag-phase of 25 nM sAβ oligomerization from 2 to 3 h and slowed the rate of oligomer formation, whereas only 50 nM BR reduced the oligomer size at plateau (RH ∼ 75 vs. 110 nm) (Figure 10A and Table 1, One-way ANOVA followed by Tukey’s test, ^∗∗^*P* < 0.01 vs. sAβ). With regard to 6.25 nM fAβ, neither 50 nM CORM nor 50 nM BR affected the rate of fibril elongation, whereas this latter reduced fibril RH at plateau (RH ∼ 209 vs. 267 nm) (Figure 10B and Table 1, One-way ANOVA followed by Tukey’s test, ^#^*P* < 0.01 vs. fAβ). Selected experiments have excluded any significant effect of ruthenium, contained in CORM-2, and residual DMSO on ROS generation as well as Aβ oligomer formation and fibril elongation (data not shown).

**TABLE 1:** Effects of carbon monoxide donor CORM-2 and bilirubin (BR) on β-amyloid (Aβ) aggregation.

Treatment	Maximal RH	*p*
	(nm)	(hours^–1^)
sAβ (25 nM)	110 ± 5	0.861 ± 0.128
+ CORM-2 (50 nM)	98 ± 4	0.460 ± 0.040**
+ BR (50 nM)	75 ± 4**	0.273 ± 0.035**
fAβ (6.25 nM)	267 ± 4	0.202 ± 0.013
+ CORM-2 (50 nM)	266 ± 4	0.244 ± 0.013
+ BR (50 nM)	209 ± 6^#^	0.180 ± 0.012

fAβ, fibrillar Aβ; nm, nanometers; RH, hydrodynamic radius; sAβ, soluble Aβ. Data are expressed as mean ± SEM (*n* = 3) and analyzed by One-way ANOVA followed by Tukey’s *post hoc* test: **P < 0.01 vs. sAβ and ^#^*P* < 0.01 vs. fAβ.

These last results show an unprecedented direct effect of both CO and BR on Aβ aggregation independent of the modulation of intracellular signaling pathways acting downstream. The gaseous nature of CO and the high liposolubility of BR may have a role in their direct interaction with Aβ over the transition through the structural states.

## Discussion

The role of HO-1 in the pathogenesis of AD and its druggability are no longer matter of debate. Over the years, several research groups described a marked induction of HO-1 in *postmortem* brain tissues as well as in plasma and lymphocytes from patients with AD or mild cognitive impairment (MCI), this latter being the transitional phase from healthy aging to AD (Calabrese et al., 2006; Di Domenico et al., 2012). The rationale to explain HO-1 induction in AD is related to the neuroprotective features of this early gene/protein whose ability to prevent heme toxicity, enhanced during excessive free radical generation, and to release the antinflammatory gaseous molecule CO (Nitti et al., 2018), make this enzyme a pivotal player in the cell stress response (Mancuso et al., 2013; Motterlini and Foresti, 2017). However, a large slice of literature focused on the potential neurotoxic effects of a sustained HO-1 induction due to the accumulation of its by-products, but these two hypotheses were recently reconciled by keeping in mind the “dual” nature of HO-1. In this light, the potentiation of HO-1 up-regulation by drugs (e.g., atorvastatin) and nutritional herb-based agents (e.g., ferulic acid, rosmarinic acid) detected in several *in vitro* and *in vivo* preclinical models of free radical-induced diseases, is currently considered an effective neuroprotective response (Calabrese et al., 2008; Butterfield et al., 2012; Catino et al., 2015; Fetoni et al., 2015).

Another important issue to be focused is the range of concentrations of sAβ and fAβ used to up-regulate HO-1. In many papers, to achieve a marked increase in HO-1 level were necessary high concentrations of Aβ, in the range 10—20 μM, whereas in our study a significant HO-1 over-expression was detected as low as 25 nM sAβ and 6.25 nM fAβ, these concentrations being close to those detected in AD brain which are well below 1 μM (Nag et al., 2011 and references therein). These findings favor the translational application of our results and support the hypothesis of an early induction of HO-1, even in the absence of an excessive deposition of Aβ as occurs in the later phases of AD. On the other hand, CXB (10 μM) has been shown to exert protective effects through the activation of HO-1 in human arterial and venous endothelial cells (Al-Rashed et al., 2018). The dose of CXB was chosen taking into account not only the drug-protein binding properties, but also based on the maximum plasma concentration, which has been reported to range approximately 2–8 μM in selected dosing regimens established in preclinical and clinical studies (Davies et al., 2000; Paulson et al., 2000, 2001).

Most of the research carried out with the purpose to study the involvement of HO-1 in AD, produced descriptive studies without addressing either the molecular mechanism(s) through which sAβ or fAβ regulate HO-1 expression or whether HO-1 modulation mitigates Aβ-induced brain injury. Our study supports the hypothesis that sAβ has a minor role in HO-1 regulation, whereas a marked induction of HO-1 has been detected as early as 24 h, a time-point long enough to allow the formation of Aβ oligomers. These last findings agree with previous studies on HO-1 induction by the Butterfield’s group who incubated sAβ for 24 h in PBS before treating gerbil synaptosomes and rat cortical neurons (Sultana et al., 2005; Perluigi et al., 2006). Our data are also in good agreement with those by Cui et al. (2019) who detected a significant HO-1 over-expression in BV-2 microglial cells by using a purified preparation of Aβ oligomers. As far as the contribution of fAβ to HO-1 regulation, our results in SH-SY5Y cells corroborate a significant induction of HO-1 and agree with the few studies carried out in *postmortem* brain senile plaques and neurofibrillary tangles (Smith et al., 1994; Schipper et al., 1995). Although the transition from monomers to oligomers and fibrils has to be considered a *continuum*, since the three Aβ aggregating forms coexist in the brain over the natural history of AD, this set of experiments confirms that a different degree of HO-1 induction occurs over the transition from soluble to insoluble forms of Aβ. Intriguingly, the ability of CXB to further enhance HO-1 protein in SH-SY5Y is maintained regardless of the Aβ soluble or insoluble species (Figure 3). This evidence suggested interpreting the potential neuroprotective effect of CXB by evaluating not only the degree of HO-1 over-expression, but mainly the antioxidant outcomes. Definitely, HO-1 blockade fully reverted both CXB-related inhibition of ROS generation and lipid peroxidation damage in SH-SY5Y cells treated with sAβ, whereas in those exposed to fAβ the inhibition of HO-1 only partially counteracted CXB-related antioxidant effect and did not affect lipid peroxidation damage (Figures 7, 8). These results strongly support the evidence of a major neuroprotective effect of CXB, through HO-1 induction, in sAβ-exposed SH-SY5Y cells.

The HO-1-dependent neuroprotective effect of CXB sheds new light on a drug whose efficacy in AD has long been debated. As early as 1997, many epidemiological studies revealed as NSAID treatment was associated with decreased risk to develop AD by reducing COX-dependent neuroinflammation. Among the determinants of this therapeutic effect, were both the type of NSAIDs used and the age of patients: the neuroprotective effect was greater with non-aspirin drugs (e.g., ibuprofen, sulindac, diclofenac) and in younger subjects (Imbimbo et al., 2010). However, these promising results were not confirmed by *ad hoc* designed clinical trials; a systematic review and meta-analysis by Miguel-Alvarez et al. (2015) has confirmed the lack of efficacy of NSAIDs, including the COX-2 inhibitor CXB, to improve cognitive skills and reduce disease severity in AD subjects. With regard to CXB, the randomized clinical trials studying its efficacy in AD enrolled either people older than 70 years with a family history of AD or patients with mild-to-moderate AD aged ≥ 50 years (Soininen et al., 2007; Group et al., 2008, 2009). That said, although not confirmed by published results, it is possible to argue that both aged subjects and AD patients recruited in these studies had already developed brain injury, due to Aβ aggregation/fibrillation, earlier than or during the clinical trials and this could be responsible, at least in part, for the lack of efficacy of CXB. Accordingly, Breitner et al. (2011) followed-up the ADAPT protocol for two additional years and found a significant reduction in AD incidence among the asymptomatic enrollees treated with NSAIDs and concluded that the efficacy of NSAID treatment depends on the stage of AD development being more effective during the earliest stage of the disease.

As far as the effectors downstream of HO-1 activation, the neuroprotective effects of CO and BR, through the down-regulation of pro-oxidant systems or direct free radical scavenging, respectively, have been extensively addressed (Piantadosi, 2008; Jansen and Daiber, 2012). Our results confirmed the ability of CO and BR to inhibit both sAβ- and fAβ- induced ROS formation and provided novel evidence for a direct effect of CO and BR in AD through the slowdown of the growth rate of Aβ oligomers and decrease in the oligomer/fibril final size. These results, *vis-à-vis* with those by Kim et al. (2019), who described the inhibitory effect of CO on the NF-κB-mediated BACE1 transcription, and by Barone et al. (2012a), who showed a strong relationship between BVR activation and BACE1 inhibition, confirm the neuroprotective role of a mild up-regulation of the HO-1/BVR system through both CO and BR. However, due to their chemical features, CO being a gas and BR a lipophilic molecule (Mancuso, 2017; Motterlini and Foresti, 2017), their potential effects on extracellular Aβ cannot be excluded.

The preclinical results described in this study parallel the clinical evidence mentioned above and put forth the adjuvant neuroprotective effect of CXB in patients with mild AD or in MCI subjects.

## Data Availability Statement

The original contributions presented in the study are included in the article, further inquiries can be directed to the corresponding author.

## Author Contributions

EM, MP, LT, and CM: conception and design of the work. EM, FP, AS, RR, VP, GP, and AF: study conduct and acquisition and of the data. EM, AS, RR, VP, GP, LT, and CM: statistical analysis and interpretation of the data. EM, MP, and CM: drafting manuscript. All authors: revising manuscript content and approving final version of the manuscript.

## Conflict of Interest

The authors declare that the research was conducted in the absence of any commercial or financial relationships that could be construed as a potential conflict of interest.
